# Abdominal Wall Reconstruction after Flap Surgery and the Effect on the Immune System

**DOI:** 10.1155/2017/2421585

**Published:** 2017-10-11

**Authors:** F. Popa, A. V. Georgescu

**Affiliations:** Department of Plastic Surgery and Reconstructive Microsurgery, Iuliu Hatieganu University of Medicine and Pharmacy, Cluj-Napoca, Romania

## Abstract

**Background:**

The aim of our study was to investigate the impact of abdominal wall reconstruction surgery on tissue anatomy and to explore how flap surgery influences the patient's immune status.

**Methods:**

Experimental abdominal wall defects were created in 8* Sus scrofa* (swine) animal models. The animals were divided into two groups: 4 swine were euthanized one month after surgery for the biopsies retrieval purpose and the other 4 swine were kept alive and the collection of blood samples has been done 6 months after surgery. In order to evaluate the relative gene expression in operated-on animal cohorts we compared them with samples from 4 healthy swine used as controls.

**Results:**

The inflammatory process was present in all types of repairs. Collagen I deposition was higher in the flap repairs. The expression level for the genes related to immune response after 6 months from surgery was relatively similar to the control group except minor alteration registered in the case of two swine models.

**Conclusion:**

Our findings indicate a less pronounced proinflammatory response to surgical trauma in animal models after flap surgery. The postoperative levels of the inflammatory cytokines did not show significant differences after abdominal wall reconstruction using flap surgery.

## 1. Introduction

The surgical approach of incisional hernias requires different techniques to repair the parietal defect that includes prosthetic material such as synthetic or biologic mesh. The biological mesh is an ideal alternative to synthetic graft, mainly in case of infection [1]. Although the biologic mesh has been associated with mixed results, outcomes of the abdominal wall reconstruction (AWR) with bioprosthetics have not been well elucidated so far [2]. The full-thickness composite of the abdominal wall defects continue to be a challenge to the reconstructive surgeon, so special considerations should be made when deciding a reconstructive surgery. The surgeon is required to address the structural, functional, and aesthetic features when considering surgical options [3].

The particular abdominal wall defect as well as the health status and lifestyle of the patient are important considerations so the goal of repair is to prevent possible wound complications: infection, seroma, hematoma, fistula, skin necrosis, and recurrences.

*Sus scrofa* was used before as an animal model to study human infectious diseases [4], due to the previously mentioned genetic similarities with the* Homo sapiens* [5]. The worldwide surgical community recognizes the need for additional animal models to further develop prosthetic materials and techniques for the hernia repair [6, 7]. The small-animal hernia models fall short in addressing the loading characteristics of the human abdominal wall. Furthermore, the use of young and healthy rats for research purposes may obscure the true effectiveness of any repair, due to their robust innate repair mechanisms. The swine model approaches a true incisional hernia model and provides a realistic comparison to the human abdominal space. The model recreates both the biological response and the mechanical challenges that accompany the complex and challenging hernias.

The question was what type of repair would be more appropriate for patients with incisional hernia. An ideal animal model thus should include layers from the skin to the peritoneum to simulate the clinical scenario; otherwise no accurate immune data or functional studies can be generated [8]. Therefore, the aim of this study was to establish a reproducible swine model, to examine its feasibility, the postoperative course, the histological analysis with haematoxylin/eosin staining and collagen staining, and the gene expression levels for immune response, and to describe the technical aspects of the new flap repairs used for the reconstruction of the abdominal wall.

## 2. Materials and Methods

### 2.1. Animals and Treatments

The study was performed under the supervision of the Department of Anesthesiology and Reanimation, within the University of Agricultural Sciences and Veterinary Medicine in Cluj-Napoca, Romania. The Ethics Committee of the University of Medicine in Cluj-Napoca, Romania, approved the experimental protocol.

Between November 2015 and May 2016, this prospective study investigated a total of 12* Sus scrofa* (swine) animals, 8 with surgical interventions and 4 healthy controls. Out of the 8 swine with surgical interventions, 4 swine were sacrificed one month after surgery. The biopsies were obtained during a subsequent necropsy while the pathological examinations were performed using hematoxylin-eosin. Collagen I : collagen III ratio was analyzed using Sirius Red/Fast Green evaluated under cross-polarized light. The other 4 swine were kept alive and the collection of blood samples was performed 6 months after surgery. After collection samples were stored on ice until gene expression for immune response was determined. In order to evaluate the relative gene expression in operated-on animals we compared them with samples collected from 4 swine that had no surgery (healthy controls). All swine were fasted overnight before the experimental procedure. The anesthesia was performed with atropine sulfate 0,04 mg/kg SC, azaperone 2 mg/kg IM (Stresnil-Janssen Pharmaceutica, Belgium), diazepam 0,1 mg/kg IM, and ketamine 10 mg/kg IM (Vetased-SC Pasteur Filiala Filipesti SRl, Romania). This protocol of sedation allowed safe transportation to the preparation room. The Propofol was administered to induce general anesthesia (1-2 mg/kg IV), Norofol (Norbrook Laboratories Limited, Northern Ireland). The swine were intubated with a straight laryngoscope blade using a number 5.5–6 endotracheal tube. To maintain the anesthesia, the isoflurane was delivered in 100% oxygen (Dräger Fabius Plus XL, Dräger Medical AG & Co, Germany). The ECG, the respiratory rate, the oxygen saturation, the pulse, the esophageal temperature, the ETCO_2_, the FiO_2_, and the anesthetic gas concentration were monitored throughout the surgical procedure. Meloxicam 0.4 mg/kg IM/24 h, Loxicom (Norbrook Laboratories Limited, Northern Ireland), was administered for pain relief.

### 2.2. Surgical Procedures

*Creation of the Abdominal Wall Defect*. A 5-cm wide × 10-cm long, semicircular, full-thickness laparotomy incision was made using surgical steel blade number 23, through the fascia of the midline linea alba and the peritoneum sack immediately below the transversalis fascia. All the conventional flaps used for the abdominal wall repair were deepithelialized.

*Flap Repairs for the 4 Swine That Underwent Necropsy One Month after Surgery*

1. Deep inferior epigastric artery perforator propeller flap (swine 1): the flap is designed as an island flap based on a single perforator, in our case the DIEP, and it is rotated 90 degrees to cover the defect.
2. The onlay technique (swine 2): this represents the repair in which the polypropylene mesh is placed over the abdominal wall closure in the subcutaneous prefascial space.
3. Advancement flap (swine 3): this is a procedure that involves the linear advancement of the deepithelialized flap in one direction.
4. Perforator “plus” flap (swine 4): this method combines the identification and the isolation of a patent perforator (the subcostal artery perforator) through a careful dissection by retaining also its base in continuity with the donor-site, which improves both the arterial supply and the venous drainage.

The biopsies of the abdominal wall repair were gathered during the necropsy, one month after abdominal wall repairs. The specimens were preserved in 10% formalin, embedded in paraffin, sectioned to 5 *μ*m, and stained with hematoxylin-eosin and Sirius Red/Fast Green.

*Remodeling Characteristics*. Masson's trichrome and the hematoxylin-eosin- (H&E-) stained slides were used to identify remodeling characteristics. One single pathologist evaluated under light microscopy the slides of each specimen.

*Collagen Distribution*. The Sirius Red/Fast Green-stained slides were prepared and evaluated according to the methods presented previously [8]. Each slide was photographed under cross-polarized light using an Axioskop 40® microscope (Carl Zeiss®) equipped with a Zeiss Axiocam® at 400x magnification. The Axiovision 4.7® (Zeiss®) software was used to semiquantitatively evaluate both areas (*μ*m^2^) that appeared red under cross-polarized light (for collagen I) and the areas that appeared green under cross-polarized light (for collagen III) on each slide.

*Flap Repairs for the 4 Swine (Kept Alive) on Which Immune Status Was Investigated Six Months after Surgery and Compared to the Gene Expression from the Healthy Controls*

(5) *The DIEP + Mesh repair *(P1) was designed as an island flap in our case DIEP, which was fixed into the defect and supplemented with a lightweight polypropylene mesh, in the onlay position, extended beyond the line of the closure by 3 cm in all directions.
(6) *The Filatov-Gillies tubed pedicle flap *(P2) is based on cutting a quadrangle flap which remained attached to the long axis representing the nutrient pedicle and the opposite extremity was directly applied into the defect.
(7) *The transposition flap *(P3) is designed as a rectangular flap that rotated on a pivot point.
(8) The reconstruction of abdominal wall defect was performed using* Colson flap-graft *(P4).

### 2.3. RT-PCR Analysis

*Sample Collection*. The collection of blood samples was performed 6 months after surgery. After collection, the samples were stored on ice immediately for gene expression evaluation, in order to assess the relative gene expression in operated-on animal models versus healthy controls (no surgery).

*RNA Extraction, Quality Control, and Assessment of Gene Expression*. For the extraction of total RNA, we used the Trireagent (Sigma-Aldrich) protocol, and the quantitative and qualitative control was performed using the Nanodrop-1000 spectrophotometer (Thermo Scientific) and the Agilent Bioanalyzer 2100. The cDNA synthesis was performed using the High Capacity cDNA Reverse Transcription Kit (Applied Biosystems) and the SuperScript III Reverse Transcription (Invitrogen), according to the manufacturer's recommendations. For the qRT-PCR, we used the SYBR Select Master Mix and the VIIA7 instrument from Applied Biosystems. Specific primers were used for each gene of interest (Tables 1 and 2). The alterations of the gene expression levels for the studied transcripts were evaluated using the ΔΔCt method.

## 3. Results

The criteria for special reconstruction techniques include performing on large defects, in absence of stable skin coverage, the recurrence of the defect after prior closure attempts, any infected or exposed mesh, systemic compromises, local tissue compromises (irradiation, corticosteroid), or concomitant visceral complications (enterocutaneous fistula). In the literature these types of flaps are used for face, hand, and leg reconstruction. This is the first study that highlights the results of the reconstruction of the abdominal wall using these types of flaps.

To begin, the histological features of a part of subsequent reconstructive surgery will be described in order to illustrate “normal histological features” and used as reference, followed by an account of a range of pathological lesions in the healing processes seen in each repaired model.

### 3.1. Histological Features

**Figure 1:**
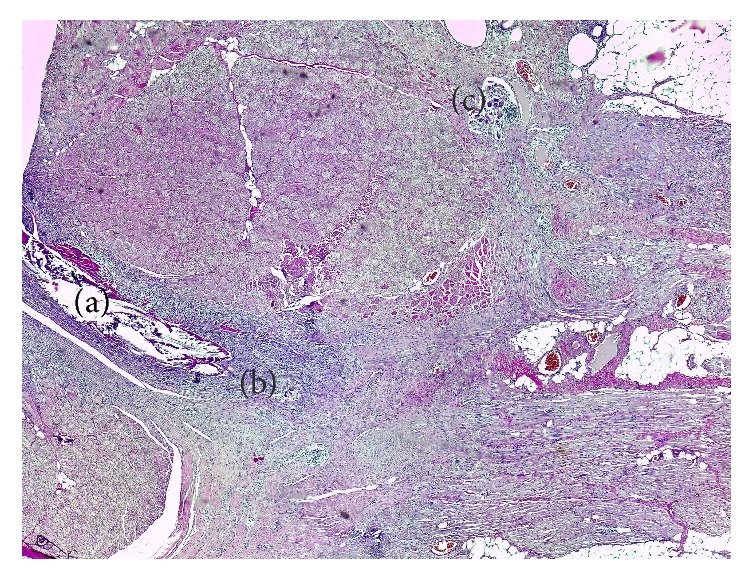
Flap H&E-stained section: (a) suture wire; (b) the foreign body cells near the flap; (c) the resorption of the suture thread by the foreign body giant cells.

**Figure 2:**
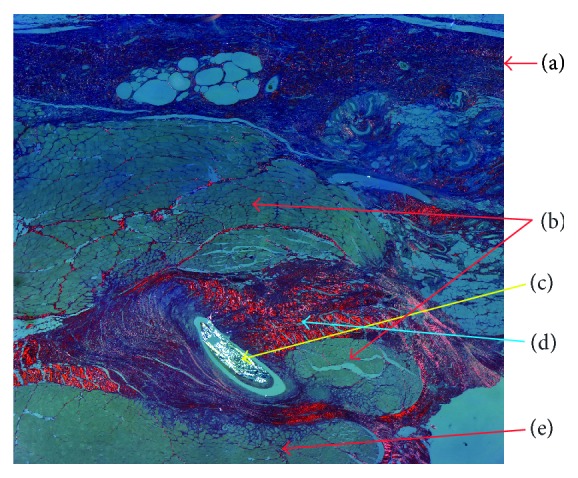
Sirius Red/Fast Green-stained slide under polarized light: (a) superficial fascia; (b) flap; (c) suture wire; (d) tendon conjunctive tissue; (e) abdominal wall muscle layer.

*For the first swine* repaired by using the deep inferior epigastric artery perforator propeller flap, the H&E-stained slides show the perpendicular orientation of the fasciocutaneous flap fibers compared to the fibers direction of the abdominal wall muscles layer. Foreign body giant cells (Figure 1) were present, which indicate the resorption role of the suture thread. The flap is positioned between the superficial fascia and the abdominal wall muscles layer. It is surrounded by a fibrinous granulosum reaction that forms a capsule around it. The fibrinous encapsulation is the result of the inflammatory process induced by the flap in the subfascial adipose tissue. The Sirius Red-stained area evaluated under cross-polarized light shows a conjunctive capsule with a tendon structure formed around the flap and suture wires (Figure 2).

**Figure 3:**
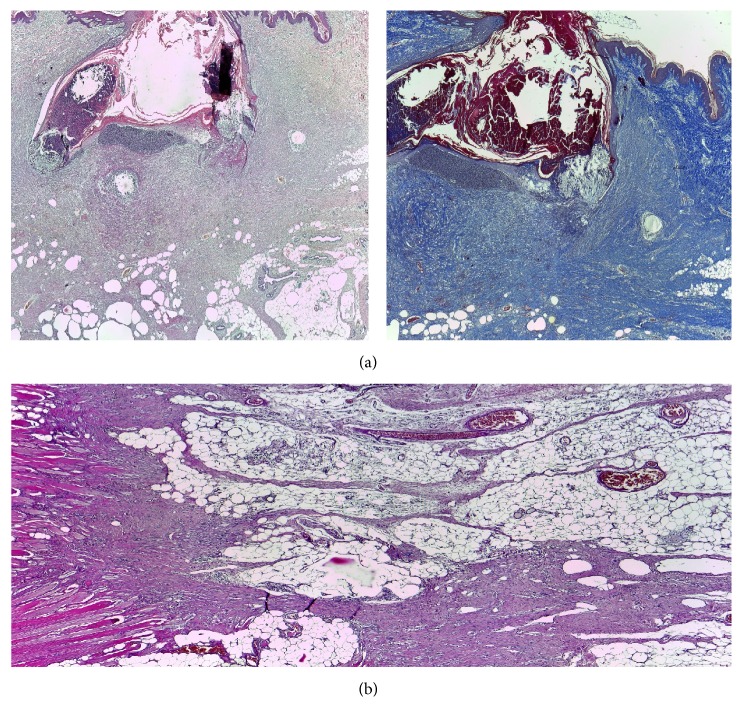
Flap H&E-stained section: (a) the inflammatory process as a diffuse panniculitis; (b) the muscular layer which was destroyed and replaced with a type of panniculitis septal fibrosis.

At the H&E evaluation of the* advancement flap repair* biopsy, the suture wires were encapsulated at the surface. An inflammatory process in the form of a diffuse panniculitis was extended from the dermis to the aponeurotic fascia with an irregular pattern. The muscular layer was deeply destroyed and replaced with a type of a panniculitis septal fibrosis (Figure 3). In Sirius Red stains the muscle fibers are interrupted and continue with a fibrosis, which is anchored in the adipose tissue.

**Figure 4:**
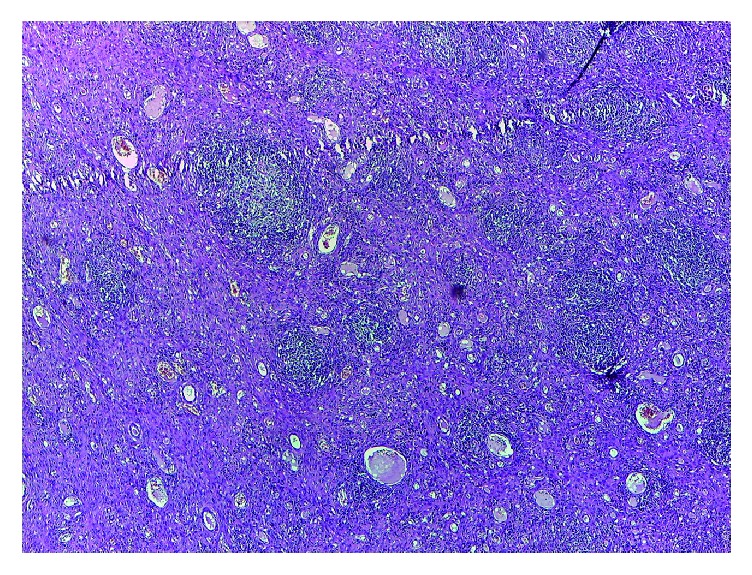
Microabscesses formed in the fibrous mass that completely replaced the fat tissue.

In the* perforator “plus” flap model* an inflammatory process type panniculitis destroys the adipose and muscle tissue and it is converted into a scar tissue which is rich in collagen I. Numerous microabscesses were created in the fibrous mass, which completely replaced the fat tissue (Figure 4).

**Figure 5:**
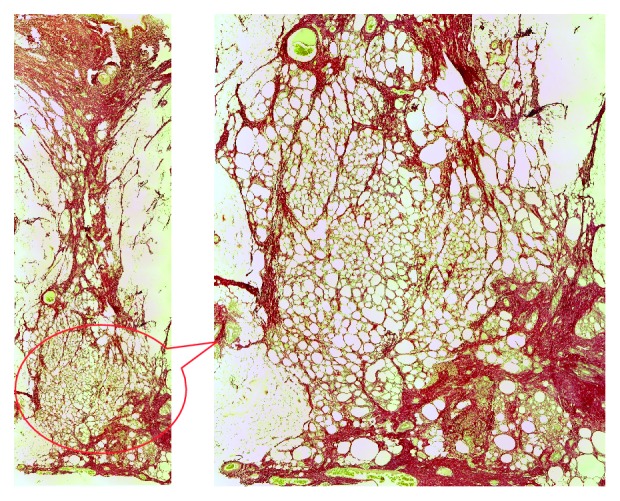
Conjunctive bridge between the dermis and the aponeurosis. The detail on the right side shows the septal panniculitis.

The mesh infection was detected in the* only model*, therefore between the aponeurotic fascia and dermis where a conjunctive bridge (scar) was formed strewn with small abscesses containing encapsulated prosthetic material (wires arranged in pus).The septal panniculitis has formed the septum by an inflammatory process (Figure 5).

### 3.2. Collagen Distribution

The Sirius Red-stained area evaluated under cross-polarized light further quantifies the collagen I surface area and collagen III surface area (Table 3). Because of the fibrosis achieved by the panniculitis and the fibrous encapsulation of the permanent synthetic mesh, the swine model in which mesh repair was applied does not contain collagen I. Therefore it has no mechanical resistance. For the advancement flap, the Sirius Red-stained area evaluated under cross-polarized light reveals a fibrosis that is dense and rich in collagen I. Therefore the repair process is resistant. The perforator “plus” flap model likewise shows a dense network of collagen I.

### 3.3. The Gene Expression Levels for Immune Response Quantification by qRT-PCR

**Figure 6:**
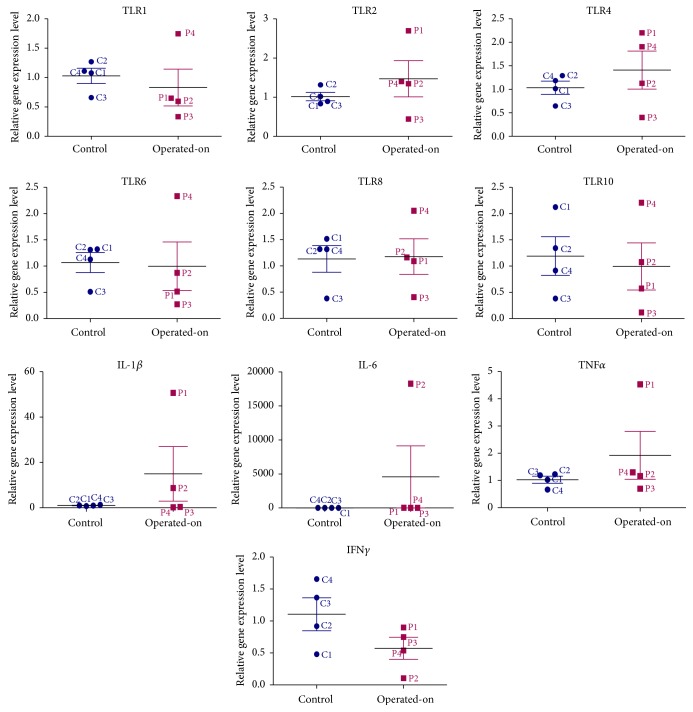
Fold change for the immune response with the related genes.

There was no significant difference between the operated-on swine models and control group regarding gene expression level. As we can see in Figure 6, several minor differences (TNF-L, IL-1B, and IL-6) were observed in the immune response in two swine models (DIEP + Mesh repair-P1 and Filatov flap repair-P2), but these need to be validated on a higher number of animals.

## 4. Discussion

Literature provides us with multiple information concerning the reconstruction of the abdominal wall but the novelty of this experimental research lies in the selection of using these particular fasciosubcutaneous flaps.

This study highlights the new flap techniques used to repair the abdominal wall defect and provides readers with a better understanding on the postoperative course, the flaps histology, and the immune consequences. The pigs have been utilized in numerous studies of tissue healing and blood flow since they mimic human cutaneous and vascular anatomy [9–12]. The animal models offer important advantages for plastic repair progress. The present study tests the applicability of the flaps (commonly used in plastic surgery to repair facial, hand, and foot defects) used for the reconstruction of the abdominal wall hernia on swine and examines changes in the inflammatory response compared to healthy swine who had no surgery used as controls.

### 4.1. Flap Selection for Hernia Repairs

The flap coverage proposed in our study for the reconstruction of the abdominal wall was done using fasciosubcutaneous flaps, which are widely used in hand reconstructive surgery.

They can be grouped in

- *pedicled flaps* (advancement, transposition, Filatov-Gillies),
- *perforator flaps* (DIEP, perforator “plus”),
- *skin grafting* (Colson).

The reconstruction is typically performed in a pedicled fashion because it leads to minimal complications and it is easy to perform it. Because their blood supply is provided through short perforator pedicles, they have to be harvested in the immediate vicinity of the defect. Minimal dissection is needed to gather the flaps, having a broad base of implantation. When using the pedicle option, it is important to pay attention to venous congestion to not compress the flap or pedicle.

By gathering these kinds of flaps, it is also possible to negatively influence the postoperative evolution by developing a more or less transitory edema or hematomas, which may compromise the local flap vascularity by inducing vasospasm, stretching the subdermal plexus, or separating the flap from the surface of the recipient site. Vascular damage can be produced by the difficulty in handling the perforator vessel, the extreme compression, or the devascularization of the area.

When the conditions of the wound and surrounding tissues of the defect do not allow the use of pedicled flaps, it is known that perforator flaps have a major advantage due to the arc of rotation. In our cases, the propeller perforator flaps which were rotated 90 degrees had a good evolution, which makes this option worth being considered.

The Colson flap can provide cutaneous coverage of the abdominal wall defects. Given that skin grafts provide the little musculofascial support but they are generally not used when there is loss of abdominal wall musculature, therefore, they are relatively contraindicated in larger and complete defects. In situations where it is clinically inappropriate to close the abdominal wall, they can be used to cover exposed bowel.

Out of the eight flap repairs, three developed ischemia; this led to superficial necrosis; therefore, it was a failure from the esthetic point of view. The demanding technique that emphasizes the identification and the isolation of a patent perforator and the handling of it (vascular complications as torsion and buckling of the vascular pedicle) can explain the bad cosmetic results for the flap repairs. One swine model (Colson flap) had a recurrence because of the lack of structural support, as we already explained. The mesh infection was reported in one swine model (the only technique). Although special attention had been paid to the manipulation and fixation of the polypropylene mesh, it was interpreted as a possible foreign body reaction or a super infection of a hematoma. If the mesh implants are unable to integrate in the body, two reactions can occur: encapsulation or rejection-situation in which the saving solution is given by the use of flaps. Indications for the need of a flap surgery in the abdominal wall reconstruction include contaminated wounds, complex repairs at high risk for developing wound healing problems, high likelihood of a cutaneous exposure, mesh infection, or rejection. Patients repair options remain the use of biological mesh and flap surgery. The biological mesh repairs are limited by the high costs, making flap surgery the available alternative. Another consideration that draws attention is the lack of literature in long-term outcomes and the contraindications for the use of bioprosthetic mesh in the abdominal wall reconstruction [13]. This is why we should ask ourselves: where do these intense indications to use biological mesh come from, instead of a flap surgery? The use of the autologous tissue in the form of pedicle and free flaps provides optimal results in the majority of these cases and it can be safely performed with knowledge of the benefits and the potential limitations of their usage [14–16]. The flap repair is widely used as a choice in poor countries for patients with wound infection, enteric fistula, or stoma, where there is a high risk of mesh infection or mesh rejection that could lead to dramatic consequences.

### 4.2. Histological Approach

**Figure 7:**
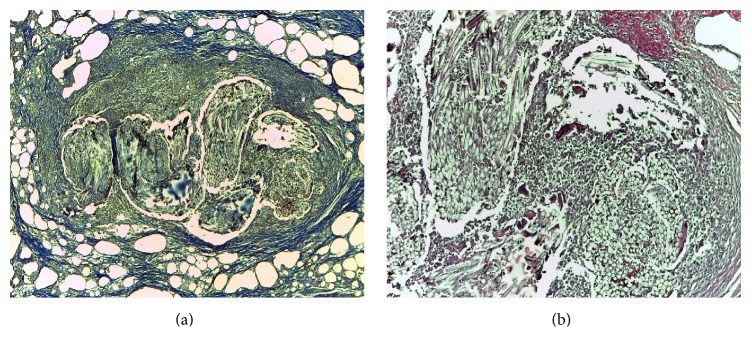
Histological view of the infected mesh: (a) Masson's trichrome stain, 40x, suture wire encapsulated; (b) H&E stain, 100x, suture wires surrounded by PNM.

**Figure 8:**
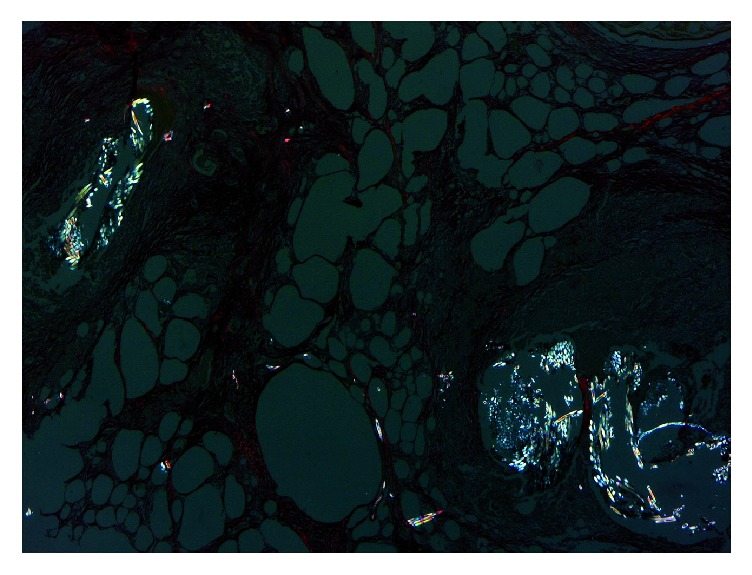
The absence of collagen I fibers seen on Sirius Red under cross-polarized light.

**Figure 9:**
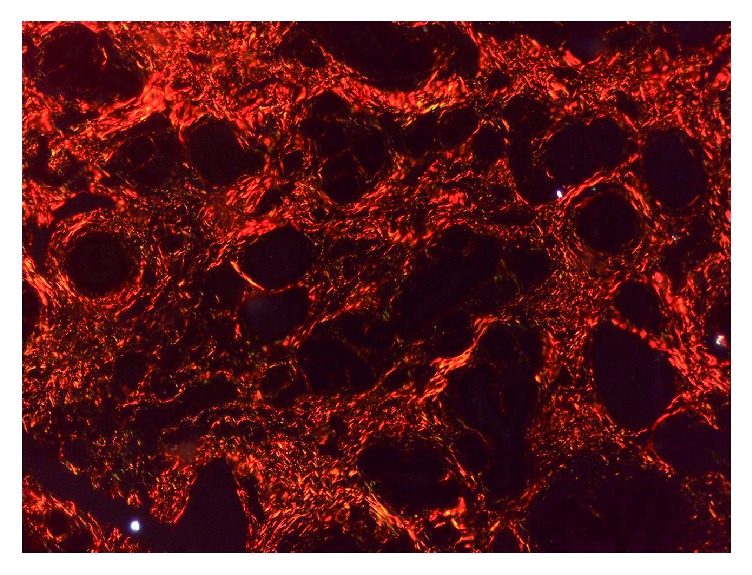
Dense network of collagen I seen on Sirius Red under cross-polarized light.

The phases of acute wound healing are described as hemostasis, inflammation, fibroproliferation (scar formation), and wound remodeling. A defect or delay in the activation of any of the repair pathways expressed during normal laparotomy and hernia repair may lead to hernia formation [17]. The paucity from the literature concerning similar studies has aroused our interest to investigate the histology of different types of abdominal wall repairs and to compare the outcomes. The flap models compared with the mesh flap model indicate a presence of cell types significantly more favorable for constructive remodeling repair. These may suggest that polypropylene fibers negatively affect the uniform cellular migration to the center of the scaffold during remodeling. Surgical sites that were “not clean” at the time of mesh explantation were associated with a lack of cellular penetrance to the center of the mesh specimen, indicating less constructive remodeling [18]. The fundamental mechanism may be an underlying wound healing defect, or an inadequate surgical technique. When an abdominal wall repair fails due to inadequate surgical technique, selective changes occur: the wound fibroblasts and the extracellular matrix molecules lead to a pathological chronic wound. Examples are represented in our study by the multiple microabscesses formed in the fibrous mass that completely replaced the fat tissue or the small abscesses containing encapsulated prosthetic material by suture wires arranged in pus (Figure 7). In terms of the delayed fibroblast responses, turns impede the synthesis of a provisional wound matrix, prolonging the period of time. A surgical wound is subjected to increased mechanical loads and depends entirely on suture material and technique for its strength. The fibroblasts are responsible for collagen synthesis and the recovery of a wound's breaking strength. The mechanism by which the collagen-rich early laparotomy wound matrix attaches to uninjured tissue at the wound border is also poorly understood [19]. The combination of Sirius Red staining and visualization under polarized light microscopy is considered highly sensitive and specific for collagen types I, II, and III [20]. Therefore, the collagen that appeared red and green in the tissues studied was collagen I and collagen III. Out of the four specimens for which the abdominal wall reconstruction was applied, on the Sirius Red staining visualized under polarized light microscopy, the main quantity of type I collagen was found in the only mesh repair sample (Figure 8). A significant increase in type I collagen was viewed on the advancement flap repair slides (Figure 9). The data indicates more favorable quantities of type I collagen for the flap repairs compared with permanent synthetic mesh repair.

### 4.3. Immune Response

The abnormal collagen metabolism plays an important role in the pathogenesis of recurring incisional hernias. Surgically, it is equally likely for these pathological changes to cause the reduction of abdominal wall compliance and to contribute to the difficulty of achieving durable incisional hernia repairs.

As we expected, the flap surgery does not affect in any way the immunity of the host. The small changes for the three genes TNF-L, IL-1B, and IL-6 recorded in our study could explain the reaction of the innate immunity to the polypropylene mesh for the first swine model, acting against a non-self-antigen detection due to inflammation. For the second swine model, the only explanation we found was that the surgical procedure is based on two surgeries (the second surgery being performed at two weeks after the first intervention). Another thing that caught our attention was the fact that this swine model was the only one who did not gain weight in the six months after surgery. At the six-month evaluation, all tests (blood and ultrasound tests) were normal for this swine model. This finding ensures surgeons that by choosing flap surgery for patients with immune problems, the surgery will not affect in any way their health status.

The patients' immunity-cicatricial condition is essential for the success of the repair. The tumor necrosis factor-*α* (TNF*α*) is a cytokine involved in the systemic inflammation and has an important role in the development of the acute phase reaction. However, this response must be closely balanced because overproduction of TNF*α* can also lead directly to apoptotic injury. Furthermore, excessive activation of NF-*κ*B can also have pathological effects, instead of protecting against apoptosis. For example, the exaggerated NF-*κ*B activation that occurs in sepsis syndromes may explain some of the pathophysiological changes, including the intravascular coagulopathy [21]. Studies demonstrate that TNF*α* induces matrix metalloproteinases (MMPs) that may disrupt skin integrity. The TNF*α* increases collagenolytic activity of MMP-1, which is probable via upregulation of MMP-3 leading to gradual loss of type I collagen in the human skin [22].

The IL-1*β* is a member of the interleukin 1 family of cytokines and is produced by activated macrophages as a proprotein, which is proteolytically processed to its active form by caspase 1 (CASP1/ICE). This cytokine is an important mediator of the inflammatory response and it is involved in a variety of cellular activities, including the cell proliferation, the differentiation, and the apoptosis [23]. This is the case of the DIEP + Mesh swine repair described in our study, where the reaction of the innate immunity to the polypropylene mesh acting against a non-self-antigen detection explained the inflammation; the rest of the animals had relatively similar results concerning the three genes TNF-L, IL-1B, and IL-6 compared with the control group.

The IL-6 is an interleukin that acts as both a proinflammatory cytokine and an anti-inflammatory myokine. The IL-6 is an important mediator of fever and of the acute phase response. It is capable of crossing the blood-brain barrier and initiating synthesis of PGE2 in the hypothalamus, thereby changing the body's temperature set point. Synergistic effects of combining IL-6 and HA (hyaluronic acid) on the cell migration of wound healing by activation of ERK and NF-*κ*B signaling were confirmed [24].

A recent study was able to establish a panel of key proteins (IL-1*α*, GM-CSF, IL-6, and IL-18) related to skin, based on combined mean-centered protein dataset, including allografts, isografts, and immunosuppressed animals. The IL-1*α*, the IL-18, the IL-1*β*, and the IL-4 were identified as principal drivers of transplant rejection [23].

IFN*γ*, or type II interferon, is a cytokine that is critical for innate and adaptive immunity against viral, some bacterial, and protozoal infections. The antitumor CD8+ T cells are a key determinant for overall survival in patients following surgical resection for solid malignancies. Studies show surgical stress results in a reduction in the number of CD8+ T cells that produce cytokines (IFN*γ*, TNF*α*, and Granzyme B) in response to TAA (adenovirus expressing melanoma tumor-associated antigen). Preoperative immunotherapy with IFN*α* significantly increases survival in surgically stressed animal models [25].

The Toll-like receptors (TLRs) represent a class of proteins that play an important role in the innate immune system. The TLR family includes TLR1, TLR2, TLR4, TLR6, TLR8, TLR9, TLR10 membrane-spanning, the noncatalytic receptors expressed in cells such as macrophages, and the dendritic cells that recognize structurally conserved molecules derived from microbes [26–29].

There were no significant differences in any serum cytokine concentration between groups: flap repairs compared to health controls at 6 months after operation, except the first swine model where the polypropylene mesh was used; being a reactogenic material it stimulates mainly the local production of proinflammatory cytokines. The local anti-inflammatory effect of polypropylene was less pronounced but persisted for longer time and was confirmed also at the clinical examination; no significant difference in serum cytokine concentration was detected between the surgical techniques sustaining the lack of activation of the immune response at 6 months after operation. Transfer of healthy tissue hinders the proinflammatory response after surgery and can explain the beneficial effects of flap procedures.

Knowing the flap techniques have no risk for graft rejection and are quick and effective procedures, which do not cause immune system changes, makes them repair options to be used for the reconstruction of the abdominal wall. This study will guide the surgeon on the new flap techniques available to be used for the reconstruction of the abdominal wall, giving him the possibility of trying or denying these types of repairs.

## 5. Conclusion

Technical, histological, and immunological aspects of the new swine model flap repairs are described leading to important surgical practical application for patients immunocompromised, confirming that it is a safe, fast, and cheap alternative for the reconstruction of the abdominal wall, which does not affect in any way patient's immunity status.

The findings reveal no difference in the immune response, following the flap abdominal wall reconstruction. The novelty of this study lies in the application of these surgical techniques for the reconstruction of the abdominal wall, creating the first experimental model of such kind of flap repairs, following its postoperative course, the immune profiles, and the histological changes. Surgeons and patients should be aware of these findings and it should be part of any discussion on the abdominal wall reconstruction procedures.

## Figures and Tables

**Table 1:** Toll-like receptor genes role and primers sequence used for qRT-PCR analysis.

Symbol	Gene name	Cellular localization and role	Primer sequence
TLR1	Toll-like receptor 1	Localized on the cell surface. Recognizing microbial components.	TGCTGGATGCTAACGGATGTC AAGTGGTTTCAATGTTGTTCAAAGTC
TLR2	Toll-like receptor 2		TCACTTGTCTAACTTATCATCCTCTTG TCAGCGAAGGTGTCATTATTGC
TLR4	Toll-like receptor 4		GCCATCGCTGCTAACATCATC CTCATACTCAAAGATACACCATCGG
TLR6	Toll-like receptor 6		AACCTACTGTCATAAGCCTTCATTC GTCTACCACAAATTCACTTTCTTCAG

TLR8	Toll-like receptor 8	Intracellular vesicles. Recognizing nucleic acids.	AAGACCACCACCAACTTAGCC GACCCTCAGATTCTCATCCATCC
TLR9	Toll-like receptor 9		CACGACAGCCGAATAGCAC GGGAACAGGGAGCAGAGC

TLR10	Toll-like receptor 10	Surface of B cells, monocytes, and neutrophils. Anti-inflammatory function.	CCTGTCCAACTGCCTCATTTG CTAAGTGTTCTAAGGATGTGTTTCTG

**Table 2:** Cytokine genes role and primers sequence used for qRT-PCR analysis.

Symbol	Gene name	Role	Primer sequence
TNF*α*	Tumor necrosis factor	Implicated in a variety of diseases, including autoimmune diseases, insulin resistance, and cancer.	CAGCCTCTTCTCCTTCCTGAT TGGGGAACTCTTCCCTCTG

IL-1*β*	Interleukin 1*β*	Inflammatory response. Involved in cell proliferation, differentiation, and apoptosis.	CAGCCAATCTTCATTGCTCA GCATCTTCCTCAGCTTGTCC

IL-6	Interleukin 6	Involved in inflammation-associated disease.	GATGAGTACAAAAGTCCTGATCCA CTGCAGCCACTGGTTCTGT

IFN*γ*	Interferon Gamma	Mutations in this gene are associated with an increased susceptibility to viral, bacterial, and parasitic infections and to several autoimmune diseases.	TCATCCAAGTGATGGCTGAA ATATTGCAGGCAGGACAACC

*β*-Actin	Housekeeping genes	With a maintenance in basic cellular function role the housekeeping genes are expressed in all organisms' cells.	GGACTTCGAGCAGGAGATGG GCACCGTGTTTGCGTAGAGG
GAPDH			ACTCACTCTTCTACCTTTGATGCT TGTTGCTGTAGCCAAATTCA

**Table 3:** Histopathological features of the flap repairs biopsies for the 4 swine models who underwent necropsy one month after surgery.

Case	Conjunctive scar	Collagen I	Collagen III	Suture wire abscess	Other abscesses	Suture wire granuloma
Swine 1	±	+	−	−	−	++
Swine 2	+	+	−	+	−	++
Swine 3	++	+++	+	++	−	++
Swine 4	+++	+++	+	+++	++	+
